# Precursors to Cholangiocarcinoma

**DOI:** 10.1155/2019/1389289

**Published:** 2019-11-13

**Authors:** Tomas Hucl

**Affiliations:** Department of Gastroenterology and Hepatology, Institute for Clinical and Experimental Medicine, Prague, Czech Republic

## Abstract

Cancers of the biliary tract include intra- and extrahepatic cholangiocarcinomas and gallbladder cancer. Biliary tract cancers are diseases with unfavorable prognoses. In recent years, several lesions have been described as precursors that precede biliary cancers. They include flat and microscopic lesions known as biliary intraepithelial neoplasia, macroscopic and tumor-forming intraductal papillary biliary neoplasia, intraductal tubular neoplasia, and mucinous cystic neoplasm of the bile duct. These conditions are rarely diagnosed, while their natural history and progression to cancer have yet to be adequately characterized. This review examines the epidemiology, pathology, molecular biology, diagnosis, and therapy of these various precursors. Further research is required if we are to better understand this evolving field and improve the prevention and early detection of bile duct cancer.

## 1. Introduction

The biliary tree consists of extrahepatic biliary ducts that enter the duodenum in the ampulla, extending proximally into intrahepatic biliary ducts. The gallbladder enters extrahepatic bile ducts via the cystic duct. The biliary mucosa is made up of a single layer of a columnar epithelium. Small oval nuclei are located at the base of cells below the eosinophilic cytoplasm. The mucosa is flat, except when present in the gallbladder where it forms villi. Located alongside the epithelium, mucous glands are drained into the lumen.

Biliary tract cancers can originate in any part of the biliary system. Representing the second most common hepatobiliary malignancy, biliary tract cancers are classified based on location: intrahepatic cholangiocarcinoma, extrahepatic cholangiocarcinoma (perihilar and distal), and gallbladder carcinoma. Of these cancers, gallbladder cancer is the most commonly observed. However, the incidence of biliary tract cancer (mainly intrahepatic) is increasing.

While gallbladder cancer is more common in women, the other biliary cancers occur predominantly in males. The geographical diversity of risk factors is responsible for major differences in the world incidence of biliary cancer. Chronic inflammation due to primary sclerosing cholangitis, hepaticolithiasis, biliary-enteric drainage, and metabolic chronic liver disease, as well as chronic biliary tract infections, including *Opisthorchis viverrini*, *Clonorchis sinensis*, HBV, and HCV, are the most significant risk factors. The highest rates are found in Asia (intrahepatic) and South America (gallbladder) [1–3].

Hepatobiliary flukes, *Opisthorchis viverrini*, and *Clonorchis sinensis*, endemic to Asia, are food-borne parasites that infect the bile duct. Patients become infected by eating raw or undercooked fish, the second intermediate host of the parasite. Their presence in the bile ducts induces chronic inflammation. Parasitic infection can be proven in the majority of ICC cases in endemic areas, with a relative risk of developing cholangiocarcinoma among carriers reported between 5 and 30 [1–3].

Primary sclerosing cholangitis results in chronic inflammation of intrahepatic and/or extrahepatic bile ducts and is the main risk factor of cholangiocarcinoma in the Western population. The lifetime prevalence of cholangiocarcinoma in PCS patients is between 5 and 15% with a yearly risk of 0.5-1.5% [4].

Choledochal cysts are rare (1/13 000 in Asia to 1/150 000 in the USA) congenital bile duct cystic dilations characterized by the presence of acutely or chronically inflamed mucosa. These cysts may be, at least in part, caused by biliary stasis and pancreatic secretion reflux from the often-present anomalous pancreaticobiliary junction [2]. The risk of developing cholangiocarcinoma, which increases with advancing age, has been estimated at around 10-15% [5]. Cancer occurs earlier than in sporadic cases and may also develop in noncystic portions of the bile duct even after cyst removal.

Gallstones are found in 70-90% of gallbladder cancers. The relative risk of developing cancer in patients with cholecystolithiasis may be as high as 23% according to some studies [6]. Nevertheless, only a small proportion of patients with gallstones develop cancer during their lifetimes. About 7% of patients with hepaticolithiasis develop cholangiocarcinoma [7].

Gallbladder polyps are elevations of the gallbladder wall that project into the lumen. According to a recent systematic review by Elmasry et al., around 70% of all polyps are pseudopolyps [8]. Nonmalignant pseudopolyps include cholesterol polyps, adenomyomatous polyps, and inflammatory polyps, whereas true polyps typically present as adenomas or carcinomas. The adenoma-carcinoma sequence in the gallbladder is not as well understood as that found in the colon. The management of gallbladder polyps is contentious. According to a recent guideline, cholecystectomy is recommended in those with polyps greater than 10 mm. Management of smaller polyps depends on patient and polyp characteristics [9].

Biliary tract cancers are highly aggressive tumors. Most patients with biliary tract cancers present at a late stage, by which time the disease is likely to have microscopically or macroscopically spread. Surgical resection, the only curative modality, can be offered to less than a third of patients, with overall 5-year survival at around 10% [2].

Due to advances in neoplasia research, precursor lesions of various cancers have gained recognition. Studies of precursors have enhanced our understanding of the origins of associated diseases and of certain developmental stages, including molecular processes that inform phenotypic changes. Furthermore, biochemical, molecular, and imaging diagnoses of precursors are being used to identify either patients at risk of developing cancer or those in the early stages of the disease as a means of providing adequate therapies and improving survival. For instance, in the pancreas, the following conditions are now acknowledged as precursors of cancer: pancreatic intraepithelial neoplasia (PanIN), intraductal papillary mucinous neoplasia, and mucinous cystic neoplasia. The latter two, which can be identified using imaging methods, are currently the subject of extensive research, with recommendations being formulated for successful management [10, 11]. All three precursors have counterparts in the biliary tract [1].

## 2. Precancerous Lesions

Based on the majority of the current literature, four types of biliary precancerous lesion are recognised. The two main precursors of biliary carcinoma are biliary intraepithelial neoplasia (BilIN) and intraductal papillary neoplasm of the bile duct (IPNB) [1, 12]. To these, the World Health Organization (WHO) 2010 classification has added mucinous cystic neoplasm (MCN) [13], with the most recent entry being intraductal tubular/tubulopapillary neoplasm (ITN) [14].

## 3. Biliary Intraepithelial Neoplasia

Biliary intraepithelial neoplasia (BilIN) is a microscopic flat or micropapillary lesion of the dysplastic epithelium [15]. BilIN and its grades represent the most frequent dysplasia-carcinoma sequence through which intra- and extrahepatic cholangiocarcinomas develop [1].

Since it is a microscopic disease, BilIN produces no symptoms and cannot be detected using current imaging methods. It is most likely found in specimens of invasive biliary tract cancer or in gallbladders resected for cholecystolithiasis. As such, its real incidence cannot be precisely determined. However, it is likely comparable to invasive cholangiocarcinoma [16]. Matthaei et al. examined resection margins of 55 biliary tree adenocarcinomas, including gallbladder cancers. BilIN was detected in the resection margins of 29 patients (53%). In the majority of cases, it was identified as low-grade (BilIN-1, 14 out of 29, 48%), with occurrence most frequent in extrahepatic carcinomas (6 out of 8, 75%). Notably, patients with positive resection margins for cancer (R1 resection) exhibited significantly shorter overall survival than those with negative resection margins (R0), irrespective of the presence of BilIN or its grade [17]. Lewis et al. examined 100 consecutive PSC liver explants, including 30 with cholangiocarcinoma, for the presence of biliary dysplasia. Cancer patients exhibited greater prevalence for all types of BilIN (83% vs. 36%, *p* < 0.0001) and high-grade dysplasia (BilIN-2 or 3, 60% vs. 11% ,*p* < 0.0001) [18]. Wu et al. investigated the presence of BilIN in large intrahepatic and hilar ducts of 244 explanted livers from patients with EtOH cirrhosis and HCV cirrhosis alongside noncirrhotic controls. Patients with EtOH and HCV cirrhosis were much more likely to harbor BilIN compared with noncirrhotics (97%, 92%, and 55%, *p* < 0.001). BilIN-3 was present in about 5% of all cases of EtOH and HCV cirrhosis but absent in noncirrhotic controls. Three out of 8 patients (36%) with BilIN-3 also had cholangiocarcinoma in another part of the liver [19].

**Figure 1:**
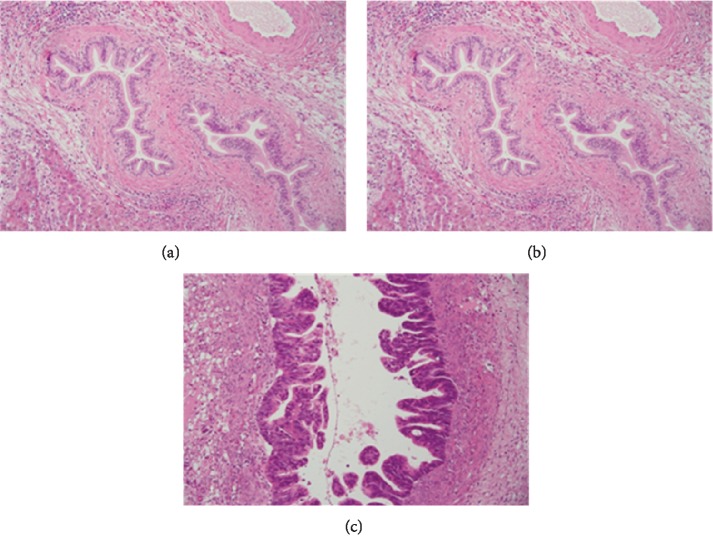
Histology image of biliary cancer precursors. (a) Biliary intraepithelial neoplasia grade 1 (BilIN-1)—bile duct with tall columnar cells with mild nuclear abnormalities. (b) Biliary intraepithelial neoplasia grade 2 (BilIN-2)—bile duct with columnar cells with moderate nuclear abnormalities and focal pseudopapillary or micropapillary architecture of the epithelium. (c) Biliary intraepithelial neoplasia grade 3 (BilIN-3)—bile duct with columnar cells with severe nuclear abnormalities and pseudopapillary and cribriform architecture of the epithelial layer. Haematoxylin-eosin staining (courtesy of Dr. Jana Maluskova).

On gross examination, BilIN can either appear normal or display subtle granularity or thickened mucosa [16]. BilIN may be totally flat or form micropapillary or pseudopapillary projections into the lumen. Microscopically, it is characterized by columnar epithelial cells with multilayering of more or less abnormal nuclei [1]. Similar to pancreatic intraepithelial neoplasia, it can be classified into three grades depending on the extent of cytological or structural abnormalities (Table 1 and Figure 1). The original criteria for each grade were established using livers of patients with hepatolithiasis [15]. However, following an international study by 17 pathologists, which revealed suboptimal interobserver agreement in respect of 30 noninvasive neoplastic biliary lesions (kappa value of 0.45 overall and 0.16 for BilIN-2); these criteria were subsequently revised [20].

In one study, analysis of microdissected BilIN for the *KRAS* and *GNAS* mutations and p53 expression of BilIN and cholangiocarcinoma revealed similarities to pancreatic intraepithelial neoplasia [10]. Existing *KRAS* mutations were observed in early BilIN-1 lesions (25%), increasing slightly during progression to BilIN-3 (30%). *GNAS* mutations were not identified in any lesions. Occurring as a late event, overexpression of p53 has been found only in invasive carcinoma (intrahepatic—18%, extrahepatic—38%, and gallbladder—62%) [21].

Variable mucin and epithelial markers have been described for BilIN lesions. Typically, BilIN lesions are characterized by MUC1 and MUC2 negativity. However, increased expression of MUC1 occurs as BilIN-1 progresses to BilIN-3 [22]. This is in agreement with tubular adenocarcinoma, typically associated with BilIN, being positive for MUC1 [23]. MUC2 positivity is rarely found, suggesting an intestinal phenotype [24].

## 4. Intraductal Papillary Neoplasia of the Bile Duct

Intraductal papillary neoplasia of the bile duct (IPNB) is a macroscopic lesion that can present in either single or multiple form. Situated along the biliary tree, it is characterized by intraductal growth of the dysplastic epithelium with mucin hypersecretion [25, 26]. Formerly known as biliary papillomas, biliary papillomatosis, and papillary adenomas, its current term is now recognised in the World Health Organization classification from 2010 [13]. It shares similarities with its pancreatic counterpart, intraductal papillary mucinous neoplasms (IPMN) of the pancreas, and can be found anywhere on the bile tree, including the gallbladder (intracholecystic papillary neoplasm) [1].

IPNB is a rare disease that accounts for about 10-15% of bile duct tumors. It is typically found in patients between 60 and 70 years of age. It is more common in Asia (Taiwan, Korea, and Japan) where it is associated with common risk factors such as hepaticolithiasis and fluke. Interestingly, while IPNB predominates among males in Western countries, it either predominates among females or is equally distributed between genders in Asia [25–27].

Macroscopically, IPNB manifests as a single or multiple yellowish papillary mass. Filling the bile duct, it can be accompanied by a cystic dilatation of the affected segment, more commonly in extrahepatic ducts. Mucin secretion in ducts can be macroscopically visible in some patients [1, 3, 12].

IPNB follows an adenoma-carcinoma sequence [28]. It can be classified as low-grade or high-grade based on the degree of dysplasia but can also manifest in an invasive cancerous form. Invasive carcinomas are found in about 40-80% of IPNBs [3, 28].

**Figure 2:**
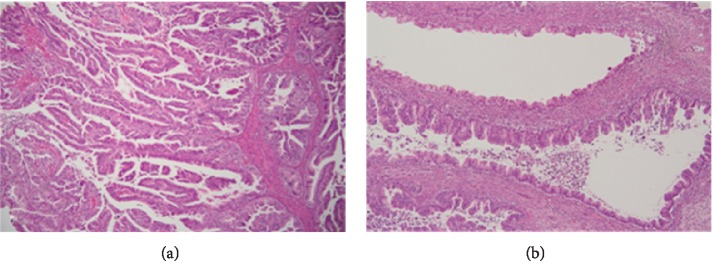
Histology image of biliary cancer precursors. (a) Intraductal papillary neoplasia of the bile duct (IPNB)—bile duct with papillary architecture of the epithelium and cytological nuclear abnormalities. (b) Biliary mucinous cystic neoplasia (BMCN)—cyst with the epithelium of the mucinous type and variable grade of nuclear abnormalities. Haematoxylin-eosin staining (courtesy of Dr. Jana Maluskova).

Like IPMN of the pancreas, IPNB is classified into four types based on histological and immunohistochemical features [27, 29] (Table 2 and Figure 2). While the pancreatobiliary type is the most prevalent lesion in Western countries, the intestinal type is most common in Asia. When IPMN becomes invasive, it typically develops into tubular adenocarcinoma, with the exception of the intestinal type, which usually progresses into mucinous adenocarcinoma [1]. It remains unclear, however, whether these histology subtypes have any prognostic relevance. In a retrospective analysis of 97 patients with IPNB, the frequency of invasive carcinoma in the pancreatobiliary type was significantly higher than in gastric and intestinal types (72.7 vs. 26.7 and 32.6%, *p* < 0.001) [30]. Accordingly, survival was significantly worse in subjects with the pancreaticobiliary type compared to those with gastric and intestinal types (*p* = 0.035). However, although these observations are similar to findings for pancreatic IPMN, they have not been confirmed by other IPNB studies [1, 28].

The progression of dysplasia is accompanied by the accumulation of genetic alterations. *KRAS* alterations occur early and precede increased expression of TP53 and loss of SMAD4. Surprisingly, *GNAS* mutations, which are linked to the molecular pathogenesis of pancreatic IPMN [31], seem to be much less important [28]. Notably, all *GNAS*-mutated IPNBs are of the intestinal subtype, similar to pancreatic IPMN [28, 31].

IPNB can be asymptomatic and found incidentally. Typical clinical manifestations include abdominal pain, most commonly in the right upper quadrant, obstructive jaundice, and cholangitis. Weight loss and anemia are signs of malignancy [3, 12].

Laboratory investigation may reveal signs of biliary obstruction. The role of tumor markers is unclear. In one study, elevation of CA 19-9 occurred in 40% of patients with IPNB [32]. In another study, CA 19-9 elevation occurred in 35% of benign lesions and 61% of malignant lesions; however, this difference was not statistically significant [33].

**Figure 3:**
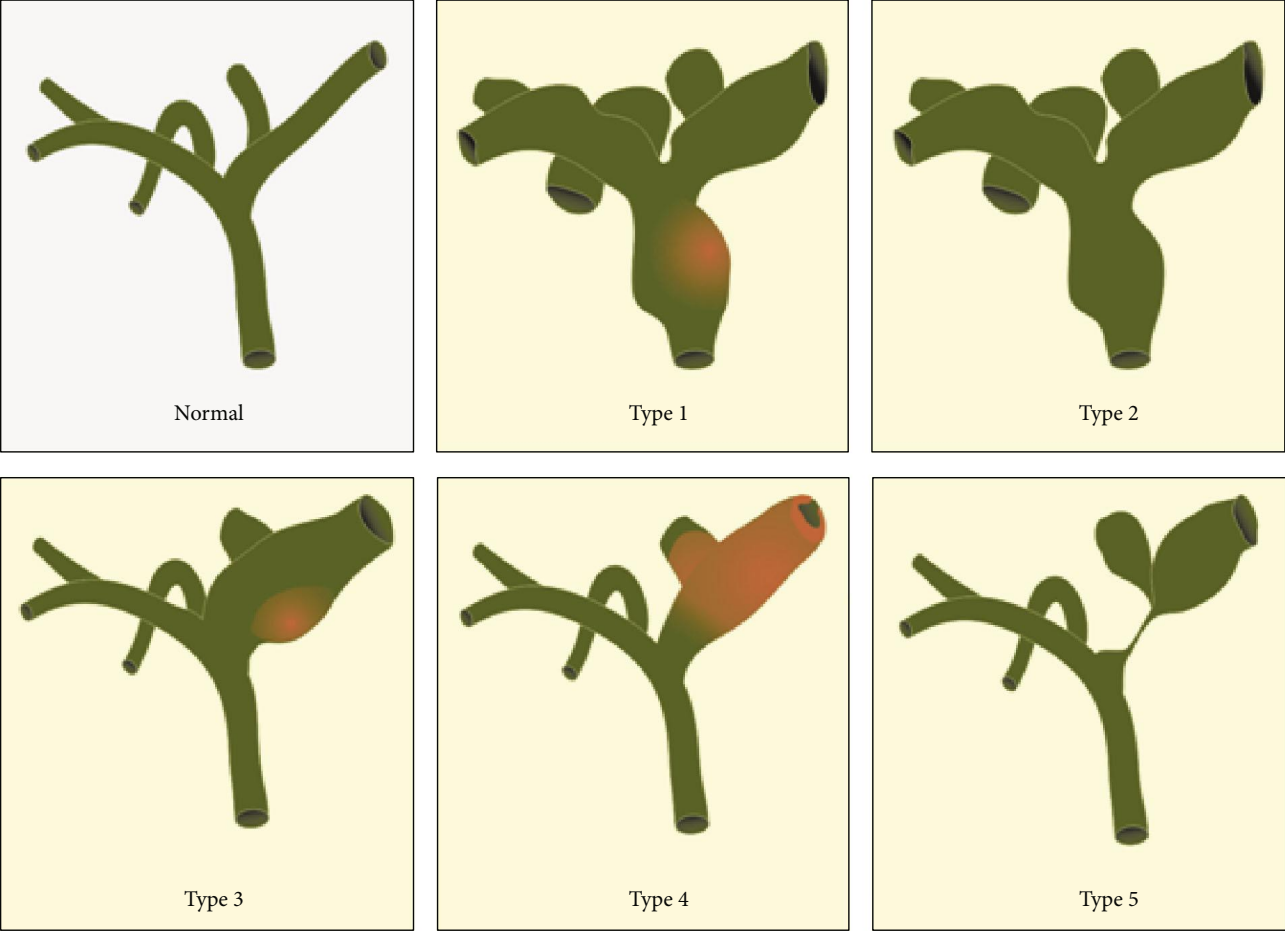
Anatomical classification of intraductal papillary neoplasms of the bile duct (figure based on Wan XS, WJG 2013).

Imaging results often show IPNB in the form of an intraductal mass and/or a dilatation of the surrounding bile duct. Kim et al. have proposed the following anatomical classification [30]: type 1—a grossly visible intraductal mass with diffuse dilation; type 2—presence of a diffuse dilatation without any intraductal mass; type 3—a localized dilatation around an intraductal papillary mass; type 4—a mild ductal dilatation filled with cast-like lesions; and type 5—a focal, stricture-like lesion accompanied by a proximal duct dilatation (Figure 3). Abdominal ultrasound can show a bile duct dilatation or a low-echoic mass in the bile duct. Endoscopic ultrasound or intraductal ultrasound can show a duct dilatation or hypoechoic mass and also evaluate signs of invasion and lymph node metastasis. CT and MR scans can detect bile duct dilatations and, generally, tumors greater than 1 cm. Cholangiography, either with MR, ERC, or PTC, can further delineate bile duct anatomy and the extent of the disease. Cholangioscopy is used for visual diagnosis, targeted biopsies, evaluating the extent of the disease, and ruling out additional synchronous lesions [3, 32]. Nevertheless, accurate preoperative diagnosis is difficult due to the rarity of the disease and the nonspecific nature of most of its clinical manifestations and imaging findings.

Surgery is the treatment of choice for those without distant spread. Preoperative staging is needed before locally advanced diseases, lymph nodes, or other distant metastases can be ruled out. Surgical treatment follows the same rules as applicable for cholangiocarcinoma and may require liver and pancreas resection [34]. Liver transplantation has been reported in individual cases [35]. In cases where surgery cannot be performed, palliative systemic therapy or local treatments may be considered [36].

Survival of patients resected for IPNB is understood to be better than of those resected for conventional cholangiocarcinoma [37]. In a study of 58 patients with IPNB, 5-year survival after curative resection was 81%, while mean survival was 60 months compared to 36 months in patients treated conservatively [32]. In a recent study by Kim et al., which documented outcomes of 112 patients resected for IPNB, positive resection margins (75.9% vs. 25.7%, *p* = 0.004) and lymph node metastasis (75.3% vs. 30.0%, *p* = 0.091) were associated with poor 5-year overall survival rates. Interestingly, histological subtypes had no effect on survival [38].

## 5. Intraductal Tubular/Tubulopapillary Neoplasia

Small case series have reported rare types of noninvasive intraductal bile duct tumor: intraductal tubular [14] and tubulopapillary neoplasms [39]. They are characterized by tubular architecture, sparse formation of papillary elements, and minimal or no mucin production [39]. In the first report, 10 patients with tubular neoplasia were examined, all presenting with obstructive jaundice and abdominal pain. Eight patients underwent surgery, and their tumors were between 0.6 and 8 cm in size. Eight cases were intrahepatic, with the remaining two extrahepatic (hilar and distal). An extraductal invasive component was present in 7 out of 9 cases [14].

In the second report, 20 cases of intraductal tubulopapillary neoplasia were compiled from various parts of the world. The mean age of patients was 62 years. Tumor location was intrahepatic in 70% and extrahepatic in 30%, with a mean tumor size of 7 cm. Necrosis was present in the vast majority of cases, with tumors staining positive for MUC1 and MUC6 and negative for MUC2. In 80% of the patients, an invasive carcinoma was present. *KRAS* mutations were present only in 6%, whereas *CDKN2A/p16* alterations were found in 44% of tumors. Despite the presence of an invasive component in most patients, 5-year survival reached 90% [39].

First described by Yamaguchi et al. [40], its pancreatic counterpart is defined as an intraductal, grossly visible, tubule-forming epithelial neoplasm with high-grade dysplasia and ductal differentiation without overt production of mucin (2010 WHO classification) [13].

## 6. Mucinous Cystic Neoplasia

Biliary mucinous cystic neoplasms (BMCN) are rare tumors characterized, like their counterparts in the pancreas, by the presence of ovarian-type stroma and the absence of communication with the biliary tree (Figure 2). They predominantly occur in females [1].

Little is known about these tumors, and because they so rarely occur, identification is difficult. Formerly referred to as bile duct/biliary cystadenoma and cystadenocarcinoma, MCN can develop in extrahepatic biliary ducts. However, as most of them arise intrahepatically, they are also called cystic neoplasms of the liver (MCN-L). They are classified based on the grade of dysplasia (low-grade, intermediate-grade, high-grade, or in association with invasive carcinoma) [1, 12].

**Figure 4:**
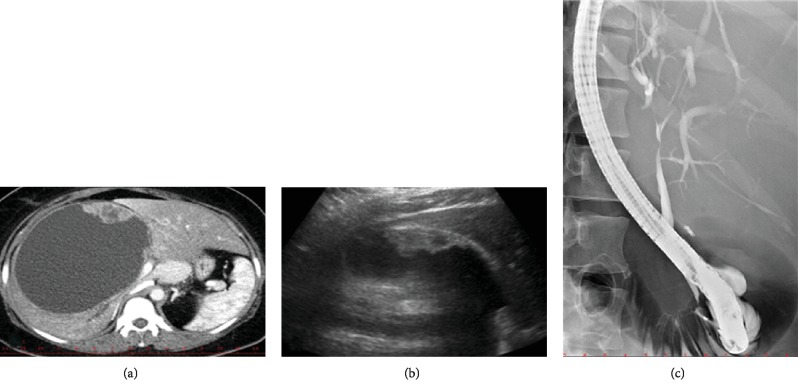
Imaging findings in a patient with biliary mucinous cystic neoplasm. (a) CT image of a large BMCN in the right liver lobe with a hypodense solid component. (b) Ultrasound image of the same BMCN with a hypoechoic solid component by the wall. (c) ERCP image of the same patient showing s smooth stricture of the hepatic duct caused by extrinsic compression of the cyst.

Albeit designated as mucinous neoplasms, the neoplastic epithelium may not produce mucus [41]. Representing only up to 5% of cystic liver lesions, they rarely occur and, when they do, typically in middle-aged women. Their origin is the subject of debate, but their most likely source is the peribiliary glands. Grossly, the neoplasms appear as a single or septated/multicystic lesion between 1 and 40 cm in size (Figure 4). Multilocular structures are more frequent. The presence of mural nodules points to malignant change. They can form a clear fluid or a thick mucus or contain blood or pus. The reported frequency of invasive carcinoma in MCN varies from 2 to 38.5% [41]. The risk of developing invasive cancer in a patient with benign MCN is estimated at around 20% [42].

Patients with MCN may be asymptomatic or present with symptoms due to a slowly growing mass, such as abdominal pain/discomfort, nausea, vomiting, obstructive jaundice, or cholangitis. Abdominal ultrasound, CT, or MRI will typically show a large cyst that must be distinguished from other more common cystic liver lesions [41]. The value of cystic fluid analysis is controversial. In one study, mucinous cysts had a higher median level of CA 19-9 compared to simple cysts (364.8 vs. 21.4 U/ml). However, no significant difference in CEA concentrations was observed (6.8 vs. 4.2 mg/l) [43]. These findings have not been confirmed in other studies [44].

Due to the high risk of disease recurrence after incomplete resection, complete R0 resection surgery is the method of choice for treating MCN. Resection or enucleation is recommended, since other interventions such as marsupialisation, aspiration, enteral anastomosis, and partial resection are associated with high complication rates [41]. Liver transplantation is another option, but early recurrence has been reported. Although dependent on the presence of invasive cancer and the completeness of the resection, the general prognosis for complete resection of a benign cyst is excellent. The 5-year survival of resected malignant MCN is 65-70%, which is better than that reported for hepatocellular carcinoma, cholangiocarcinoma, and intraductal papillary tumors [42].

## 7. Conclusions

Several precancerous lesions that precede biliary carcinoma have been reported. Even though their exact incidence is unknown, they are rarely found. Biliary intraepithelial neoplasia is an asymptomatic microscopic lesion of the dysplastic epithelium that cannot be detected by imaging. Intraductal papillary mucinous neoplasms, which can be divided into four different histological types, communicate with the biliary tree and pose a high risk of malignancy. Mucinous cystic neoplasms, which occur in middle-aged women, do not communicate with bile ducts and pose a lower risk of progression to cancer. Intraductal tubular or tubulopapillary neoplasms are rare entities similar to intraductal papillary neoplasms. However, they exhibit tubular growth and lack mucin production.

These precursors are characterized by the expression of specific mucins and follow the dysplasia-carcinoma sequence and its progressive accumulation of genetic alterations. They all have counterparts in the pancreas. However, pancreatic precursors are more frequent and better characterized, enabling more accurate preoperative diagnosis. This also means many precursors can be kept under surveillance, avoiding the need for surgery. On the contrary, not only are biliary precancerous lesions rare and difficult to diagnose, their natural history and progression to cancer are still poorly understood. As imaging methods and knowledge of the biology of biliary premalignant lesions improve, so may their detection and characterization along with the selection of appropriate treatment strategies.

## Figures and Tables

**Table 1:** Classification and characterization of biliary intraepithelial neoplasia (based on Ainechi S, *Arch Pathol Lab Med* 2016).

	BilIN-1	BilIN-2	BilIN-3
Cells	Polarity maintained Increase in basally placed nuclei	Loss of polarity Nuclei reaching the luminal surface	Marked loss of polarity Nuclei reaching the luminal surface
Nuclei	Mild atypia	Moderate atypia	Severe atypia
Architecture	Flat	Flat or micropapillary	Likely micropapillary
Mitosis	Rare	Rare	Likely
Dysplasia	Low-grade	Intermediate	High-grade/carcinoma in situ

**Table 2:** Classification and characterization of intraductal papillary neoplasms of the bile duct (based on Wan XS, *WJG* 2013).

	Pancreatobiliary	Intestinal	Gastric	Oncocytic
Histology	Columnar cells with eosinophilic cytoplasm and round nuclei	Stratified columnar cells with goblet cells	Columnar cells resembling gastric foveolae	Cells with abundant eosinophilic cytoplasm
MUC1	+	−	−	Focal +
MUC2	−	+	−	Focal +
MUC5AC	+	+	+	+
CK7	+	+	+	+
CK20	+	+	+	+
